# Components of Attention in Grapheme-Color Synesthesia: A Modeling Approach

**DOI:** 10.1371/journal.pone.0134456

**Published:** 2015-08-07

**Authors:** Árni Gunnar Ásgeirsson, Maria Nordfang, Thomas Alrik Sørensen

**Affiliations:** 1 Cognitive Psychology Unit, Leiden University, Leiden, Netherlands; 2 Cognitive Neuroscience Research Unit, Center for Functional Integrative Neuroscience, Department for Clinical Medicine, Aarhus University, Aarhus, Denmark; 3 Department of Psychology, School of Health Sciences, University of Iceland, Reykjavík, Iceland; 4 Center for Visual Cognition, Department of Psychology, University of Copenhagen, Copenhagen, Denmark; 5 Centre for Cognitive Neuroscience, Department for Communication and Psychology, Aalborg University, Aalborg, Denmark; University of Regensburg, GERMANY

## Abstract

Grapheme-color synesthesia is a condition where the perception of graphemes consistently and automatically evokes an experience of non-physical color. Many have studied how synesthesia affects the processing of achromatic graphemes, but less is known about the synesthetic processing of physically colored graphemes. Here, we investigated how the visual processing of colored letters is affected by the congruence or incongruence of synesthetic grapheme-color associations. We briefly presented graphemes (10–150 ms) to 9 grapheme-color synesthetes and to 9 control observers. Their task was to report as many letters (targets) as possible, while ignoring digit (distractors). Graphemes were either congruently or incongruently colored with the synesthetes’ reported grapheme-color association. A mathematical model, based on Bundesen’s (1990) Theory of Visual Attention (TVA), was fitted to each observer’s data, allowing us to estimate discrete components of visual attention. The models suggested that the synesthetes processed congruent letters faster than incongruent ones, and that they were able to retain more congruent letters in visual short-term memory, while the control group’s model parameters were not significantly affected by congruence. The increase in processing speed, when synesthetes process congruent letters, suggests that synesthesia affects the processing of letters at a perceptual level. To account for the benefit in processing speed, we propose that synesthetic associations become integrated into the categories of graphemes, and that letter colors are considered as evidence for making certain perceptual categorizations in the visual system. We also propose that enhanced visual short-term memory capacity for congruently colored graphemes can be explained by the synesthetes’ expertise regarding their specific grapheme-color associations.

## Introduction

A small minority of people report a consistent and automatic experience of non-physical color when presented with a grapheme. These people are called grapheme-color synesthetes, and the experience of synesthesia is described as concurrent (the subjective colors) to inducing stimuli (the graphemes). This unusual condition has captured the imagination of philosophers, neuroscientists and psychologist alike, and been a curiosity for at least two centuries [1]. In recent years, this field of inquiry has flourished into an active area of research that has accumulated of a large body of evidence on the neuro-cognitive mechanism of synesthesia. Among the most important contributions are the systematic study of visual cognition in synesthesia [2], and important studies of the dimensions in which synesthete may differ from the general population. To date, synesthetes have been associated with, among other things, elevated mental imagery [3] and creativity [4], tendencies toward certain cognitive styles [5] and better memory [6], but there is little evidence of synesthetes having neuro-cognitive functions that are qualitatively different from the general population in domains unrelated to their synesthesia.

For the present purposes, we will focus on the visual cognition of synesthetes; in particular attention and visual short-term memory. One very interesting aspect of the culminating literature on synesthesia is the relationship between synesthesia and attention [7–14] (see [2] for a review). Much of this literature focuses on the important distinction between pre- and post-categorical accounts of synesthesia; i.e. whether synesthetically induced colors emerge before or after the identity of the inducing letter is categorized [7–16]. In such studies, achromatic graphemes are presented to synesthetes to investigate how performance is affected by synesthetically induced colors, or to detect differences in brain function [17, 18] and structure [19] between synesthetes and those without synesthesia.

Less is known about the processing of stimuli, colored either congruently or incongruently, with synesthesia. Studies of grapheme congruence/incongruence effects using physically colored letters have primarily used synesthetic versions of the Stroop-task [20]. In such tasks observers are presented with a grapheme, which is colored either congruently or incongruently with regard to their synesthetic experience, and they name the color of each grapheme as quickly as possible (e.g. [21–25]). Usually, synesthetes are quicker at naming congruently colored graphemes, demonstrating the automaticity of synesthesia, and that it influences cognition even when it is task-irrelevant and harmful to performance. Yet, the results of the Stroop-tasks do not place the synesthetic interference effects at a particular stage of visual processing, nor does it specify specific attentional effects beyond general facilitation or interference. Standard Stroop-interference effects are interpreted in many different ways (e.g. [26–30]) and synesthetic Stroop-interference may be caused by other mechanisms than the standard effect. It may be the results of perceptual, lexical or decisional cognitive processing, or may even reflect the accumulated or interacting workings of multiple mechanisms. Synesthetic Stroop-tasks have shown that incongruence with a synesthetic color concurrent can slow the naming of physical colors of graphemes, but does not reveal whether the congruence or incongruence of stimuli affects the *perceptual* processing of graphemes. Furthermore, although a Stroop-tasks undoubtedly require focused attention on sequentially presented graphemes, they do not shed light on the mechanism(s) for deploying selective attention (but see [31] for a Stroop-like task that investigates the temporal deployment of attention in synesthetes).

Visual attention is to a large extent limited by two important factors; how quickly objects are processed on their way from retinal stimulation to conscious recognition (processing capacity), and how many visual objects can be kept simultaneously in visual short-term memory (storage capacity) [32]. Here, we attempted to isolate the processes of the attentional pipeline (see S1 Text), and find out which of these are affected by synesthesia congruence. We did so by using *A Theory of Visual Attention* (TVA) [33], a mathematical theory suited to modeling of both the *processing*- and *storage*-parameters in visual attention.

### The Memory of Synesthetes

Exceptional memory is probably the most salient cognitive side effect attributed to synesthesia. In the 1930’s, Alexander Luria registered the incredible memory abilities of a Russian journalist. [34]. He reported having multiple types of synesthesia, associated with different stimulus dimension, and seemingly used these experiences to support his exceptional memory for arbitrarily constructed stimulus material (e.g. grapheme matrices and meaningless mathematical equations). Baron-Cohen and colleagues [35] reported another case of synesthesia co-occurring with exceptional memory. In that case, the synesthete was diagnosed with an autism spectrum disorder and was described as a *savant*; exceptional at mental calculation and memory. Such case-reports make it tempting to propose a causal relationship between synesthesia and exceptional memory, but this is unlikely to be true. Not all synesthetes have exceptional memory, and there are certainly reports of “mnemonists” that do not report any synesthetic experiences (e.g. [36]). Nevertheless, it seems plausible that synesthesia facilitates the retention of objects in memory, e.g. by taking advantage of additional associative cues in the synesthetic experience, thereby enriching memory encoding and facilitating subsequent retrieval. Researchers have explored a link between synesthesia and memory in a number of recent studies. In one study, Yaro and Ward [37] tested 46 synesthetes and compared their memory performance to controls in various memory tasks. They provided evidence for a memory advantage in synesthetes, but this advantage was most pronounced for synesthesia congruent material and in the retention of colors. Modest memory advantages have been demonstrated in multiple studies [37–39] (see [6] for a review), but it must be noted that these advantages were nowhere near the exceptional memory abilities of the “mnemonists” described by Luria [34] and Baron-Cohen et al. [35].

#### Learning Synesthesia

Witthoft and colleagues have shown that some synesthetes can trace the origin of their grapheme-color associations to a set of toy refrigerator magnets [40, 41]. Their studies suggest that although synesthesia may only develop in people with a predisposition towards synesthesia (see e.g. [42] for an overview of genetic studies of synesthesia), the content of synesthetic experiences can be determined by associative learning of environmental stimuli. They also showed that, once established, synesthetic associations could remain consistent for several years without exposure to the original stimulus material, considering that the study tested adults that had adapted their grapheme-color associations from childhood toys. In fact, the same researchers have recently published a follow-up study of thousands of synesthetes, demonstrating that the generation of people raised in the United States during the sales-period of this popular brand of alphabet refrigerator-magnets were very likely to have many grapheme-color associations in accordance with the colors of the toy magnets. The same trend was not found in other parts of the world where the magnets were rare or unavailable [43].

Whether synesthetic associations are formed by environmental stimuli, or arbitrarily established in the mind of individual synesthetes, automaticity of synesthetic color experiences may have the potential to affect the memory stage of visual processing, e.g. the capacity of visual short-term memory (VSTM). This could occur in at least one of two ways: 1) by partial encoding of the concurrent color, or 2) by the strengthening of perceptual categories due to learning by repeated (subjective) exposure. A synesthete may have more retrieval cues available, due to the activation of a concurrent feature. Partially encoded visual objects may thus enhance retention in memory by providing category-relevant information. Secondly, learned associations [41] may themselves enhance VSTM, as suggested by some authors [44, 45]. Sørensen and Kyllingsbæk [45] investigated the capacity of VSTM in different age groups, and found that the memory capacity for stimulus categories increased across age groups with increasing training, whereas the memory for stimuli that were not overtly trained, remained stable over time. They proposed that the increase in capacity is caused by more efficient retention of stimulus materials, through an optimization of the reverberant feedback loops representing the visual object in memory [45] (see also [46] for an alternate account for the developmental effect in visual short-term memory).

### Visual Processing in TVA

TVA is a formal model of visual attention that allows quantitative estimation of a number of discrete attentional parameters. The model has successfully explained variable behavioral data by evoking relatively few parameters (see [47]; for an overview). It is a *race model* [48] of attention, and assumes that all objects in the visual field compete for representation in a limited VSTM store. According to TVA, visual information is initially matched with representations (perceptual categories) in visual long-term memory (VLTM). This process takes place in parallel for the whole visual field and returns evidence values based on the extent to which the visible objects match category representations in VLTM. Each potential categorization is assigned a processing rate, which represents the probability of being represented in VSTM following a stochastic race for encoding. TVA views VSTM as a limited store with a capacity measured in number of visual objects (cf. [32, 49–51]), and is thought to reflect the material currently retained in reverberating feedback loops throughout the visual system [45, 52]. The practical consequence of objects being encoded in VSTM is that these objects will be available for immediate report (by key-press or verbal report), while other objects are thought to be unavailable to consciousness.

It is important to separate VSTM conceptually from other memory buffers that may be fully—or partly—dedicated to retaining visual information. In the current study, VSTM refers to a passive memory that sustains visual information in the absence of sensory input (see e.g. [45, 52, 53]). VSTM is known to have a severely limited storage capacity of about 3–4 visual objects, when measured in terms of discrete stimuli (e.g. [32, 51], see also [49, 54, 55], for different accounts on VSTM capacity). This passive memory buffer can be contrasted against the workspace where memory contents are manipulated and/or rehearsed (e.g. [56], cf. [57] for a discussion on the distinction between rigid *short-term* and flexible *working* memory). To separate the effects of the VSTM buffer from more flexible memory buffers it is important to severely limit exposure duration, and to present the memory contents simultaneously, so that there is no room for recoding material from passive (VSTM) to a flexible memory (i.e. working memory). The restrictions set by the current experimental paradigm differentiates the study from previous ones on synesthesia and visual memory. Earlier research often allows observers to take advantage of flexible memory resources over longer time scales [37, 39, 58, 59], making it impossible to pinpoint the affected mechanism.

**Fig 1:**
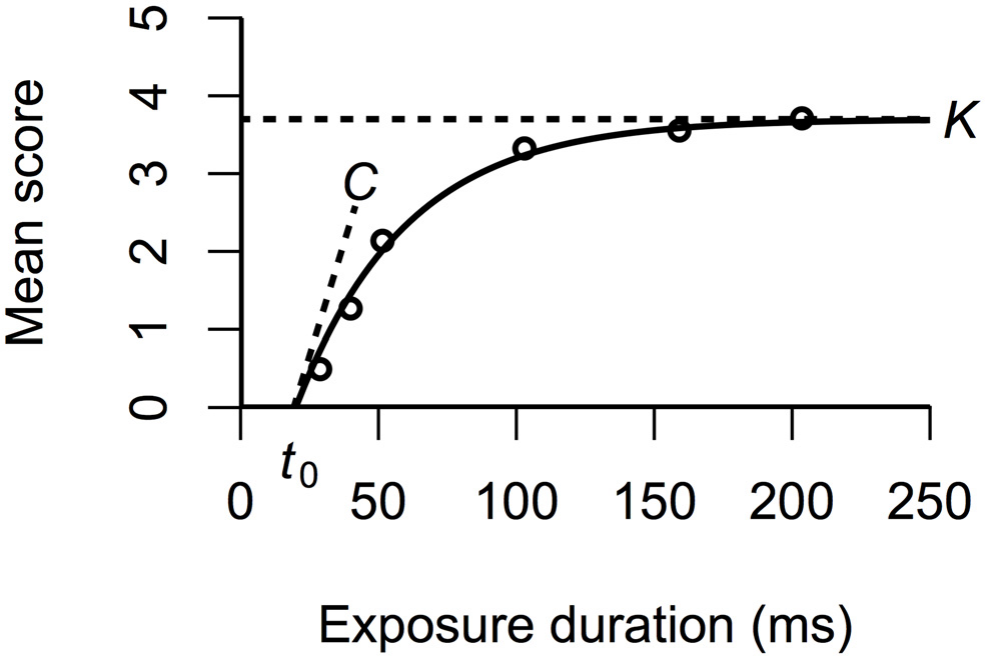
A TVA model fitted to hypothetical data. The data pattern approximates an exponential function, with a steep rise that gradually asymptotes at a certain value. This value is the estimate for VSTM *storage capacity*, or *K*. The *threshold* (*t*_0_) and *processing speed* (*C*) parameters are defined as the x-axis intercept of the fitted function and the tangent to that function at the intercept, respectively.

Fitting a TVA-model to behavioral data yields a range of specific cognitive parameters, several of which are extracted directly from these fits. When presenting an observer with an array of objects of varying durations, performance increases as a function of exposure, in a very typical pattern (see Fig 1). At very brief exposure durations, the observer will not be able to report the identity of any of the presented objects. However, as presentation times increase, so does the probability of correctly reporting one or more of the objects. This increase usually approximates an exponential function [60]. The *threshold for visual perception*, or *t*_0_-parameter, is defined at the point on the x-axis where the exponential function begins to rise.

This threshold parameter may correspond to a necessary period of spatiotemporal filtering (or pre-processing), which is needed to initiate the race towards representation in VSTM (cf. [61]). This parameter is mainly affected by the physical attributes of a stimulus, but can also be modulated by the state of the processing mechanisms (e.g. expectation [62], and pharmaceutical stimulation [63]). The parameter estimate is given in milliseconds and represents the minimum effective exposure duration. Objects presented for durations below this threshold will never be encoded in VSTM.

The rise of the performance function approaches an asymptotic value, which represents the limitations set by the storage capacity of VSTM capacity (*K*-parameter). Parameters *t*_0_ and *K* anchor the TVA-model at both ends, and an exponential function is fitted between these values. The tangent to the Cartesian point (*t*_0_, 0) in the exponential function represents the *speed of processing* (*C*-parameter). The relationship between a psychophysical function, obtained through the brief presentations of graphemes, a model fit and the parameters *K*, *t*_0_ and *C*, is illustrated in Fig 1 (see [47] for further details on TVA). S1 Text illustrates the most important aspects of attentional processing, according to TVA, in the context of the current experimental task.

To return to the terminology introduced at the beginning of the article, the presented TVA-parameters may be placed into three categories of cognitive processing. The period from stimulus presentation to *t*_0_ is determined by the *pre-processing* stage, where the physically determined sensory imprint is being integrated by spatiotemporal filtering. Given sufficient stimulation, a race will be initiated. The outcome of a race are determined by filtering (e.g. by feature or complex category) and pigeonholing into response category, which comprise the *perceptual processing* stage of processing. At this stage, attentional-biases and top-down strategies can affect the rate of processing, but the stimulus is not yet available to consciousness. The perceptual stage of processing is most generally described by the *C*-parameter (overall processing capacity), but more specifically, by the factors that determine how the resources comprising *C* are distributed; namely selectivity and bias. The *post-perceptual* stage of processing begins as soon as a stimulus is encoded into VSTM and simultaneously available for conscious report. A pre-categorical hypothesis of synesthesia should, therefore, place its primary mechanism at the pre-processing or perceptual stage, while a post-categorical hypothesis should place the mechanism at the post-perceptual stage; i.e. subsequent to recognition of the inducing stimulus.

TVA describes the workings of visual attention in two major stages that culminate in a given visual element being represented and accessible in VSTM. The first wave of processing is a massive parallel pattern recognition stage where each visual element is compared to the contents of VLTM. We refer to these contents as perceptual categories, i.e. the (rather fuzzy) collections of features that combine to make up visual objects. The result of the parallel pattern recognition wave is temporarily stored as *evidence values* that represent the degree of the match between a visual element and a given perceptual category. In the current context, the relevant categories are those representing letters and digits. We assume that perceptual categories are created and modified by experience. The sensory imprint of most stimuli will only match a few perceptual categories closely enough to evoke hard competition between categorizations (this is evident from confusion matrices; e.g. [64]). If the stimulus is very typical of its category, say an “A” written in a common typeface, the categorization may be trivial [65], due to good matches of stimulus features with the features that make up the category of “A’s”. However, if the stimulus is less typical, e.g. a handwritten cursive “A”, the stimulus features may not be a good match with any category, and the stimulus is processed less efficiently. The subjective color concurrents experienced by synesthetes may or may not be integral to the perceptual categories for the inducing graphemes. If this holds true, then a presentation of a grapheme in a congruent color should serve as evidence for that grapheme belonging to its grapheme category. A red color on the grapheme “A” should increase the evidence for that visual element belonging to the category of “A’s”, given that “redness” is a feature of the category of “A’s” in a given synesthete. According to TVA, an increase in the sensory evidence for making a certain categorization increases the rate of processing, and the likelihood of representation in VSTM. Therefore, we predicted that synesthetes would process congruently colored graphemes faster than incongruent ones. The speeded processing would be expressed as an increase in the *C*-parameter of the TVA.

In case of faster processing of synesthesia congruent graphemes, it is important to be able to discriminate between an absolute increase or decrease in processing, and a more selective allocation of resources. Under the assumption that processing capacity is a finite resource [33, 60], there are various ways of of selectively allocating resources via attentional filtering. Unique features; such as color, orientation, size and motion are filtered very efficiently [66]. However, the observers are also capable of filtering by complex categories (e.g. graphemes; [67]), albeit less efficiently. In the current context, it is quite plausible that observers will filter congruent graphemes more efficiently than incongruent graphemes. To be able to discriminate between absolute and selectivity driven advantages in processing speed, we included a selectivity parameter (*α*) in the TVA-model. This parameter represents the relative amount of resources allocated to a distractor (digit) compared to a target (letter). A selection advantage for congruent colors would therefore be expressed as a decrease in the *α*-parameter.

It has been demonstrated that VSTM holds more information when observers are experts in the stimulus material [45]. As most adult synesthetes have many years of experience with their own grapheme-color concurrent, they should be experts in their specific grapheme-color associations. Therefore, we expected synesthetes to hold more graphemes in visual short-term memory, if those graphemes were presented to them in congruent colors, i.e. matching their association of expertise. This difference would be expressed as a relative increase in the *K*-parameter of TVA for congruent letter processing.

TVA-models include a parameter which represents the perceptual threshold (*t*_0_). This threshold parameter is not thought to be affected by visual attention or higher cognitive mechanisms, but rather by physical stimulus attributes, such as contrast and spatial frequency [68] and the current state of the neural system [62, 63]. Therefore, any congruence dependent differences in the *t*_0_-parameter would indicate an important difference in the congruent and incongruent stimulus sets, or less plausibly, an effect of synesthesia at the pre-processing level.

Finally, since our hypotheses are about cognitive effects caused by synesthesia, we expected the hypothesized differences to be absent in a control group of non-synesthetes. If the control group would show processing differences dependent on the grapheme-color associations of synesthetes unknown to control participants, this would suggest that there were important physical differences between the congruent and incongruent stimulus sets.

We tested our predictions by manipulating the colors and target and distractor ratios of a mixed whole- and partial-report paradigm (e.g. [60, 69]) where observers reported the identities of briefly presented graphemes. To avoid contamination by other cognitive processes, such as decision-making and motor-control, we used an accuracy-based design with letter presentations, over a range of exposure durations (see e.g. [62, 67, 70], for similar approaches).

## Methods

### Participants

#### Synesthetes

Nine grapheme-color synesthetes (7 female) participated in the study. They were paid for their participation in gift vouchers. Their mean age was 25.8 years (*sd* = 6.1, range: 19–39 years). All were right handed. Educational level was measured on a 6-point scale from “did not finish elementary school” (point 1) to “Ph.D or equivalent” (point 6). Observers reported their latest obtained degree. The mean educational level score was 3.44 (sd = 0.73) in the experimental group, ranging from being high-school graduates to having obtained a Masters degree. The participating observers had responded to an online questionnaire where they had reported grapheme-color synesthesia. The questionnaire is available in English, Danish and Icelandic at http://www.synesthesiaproject.wordpress.com. Seven were tested at the University of Iceland and the remaining two at the University of Copenhagen.

#### Control Participants

Nine observers (7 female) participated as controls. They were compensated for their participation in gift vouchers. Their mean age was 23.4 yr. (*sd* = 2.07, range: 21–27 yr). All were right handed. The mean educational level score was 3.0 (sd = 1.0) in the control group (ranging from being elementary school graduates to having obtained a Masters degree).

Control participants were recruited through an advert on the *Center for Visual Cognition* website, and tested at the University of Copenhagen. All control observers reported no grapheme-color associations as well as normal color-vision.

#### Group Comparisons

The hypotheses in the current study regarded visual processing and memory in synesthetes and were tested by within-subject comparisons. Stimulus material was individualized for each synesthete. To ensure that any within-subjects effects measured in the synesthetes’ data, was in fact due to synesthesia and not stimulus properties, a control observer was recruited to “shadow” one of the synesthetes in the experimental group. The controls performed the experimental task with the exact same stimulus material as the synesthete they were shadowing. The same model definition was fitted to all synesthete and control participants’ data, making it feasible to see which effects, if any, are stimulus dependent, and which are driven solely by synesthesia.

All subjects signed a consent form before participation and were treated in accordance with the principles expressed in the declaration of Helsinki. The project was approved by The North Denmark Regional Committee on Health Research Ethics.

### Stimuli and Apparatus

**Fig 2:**
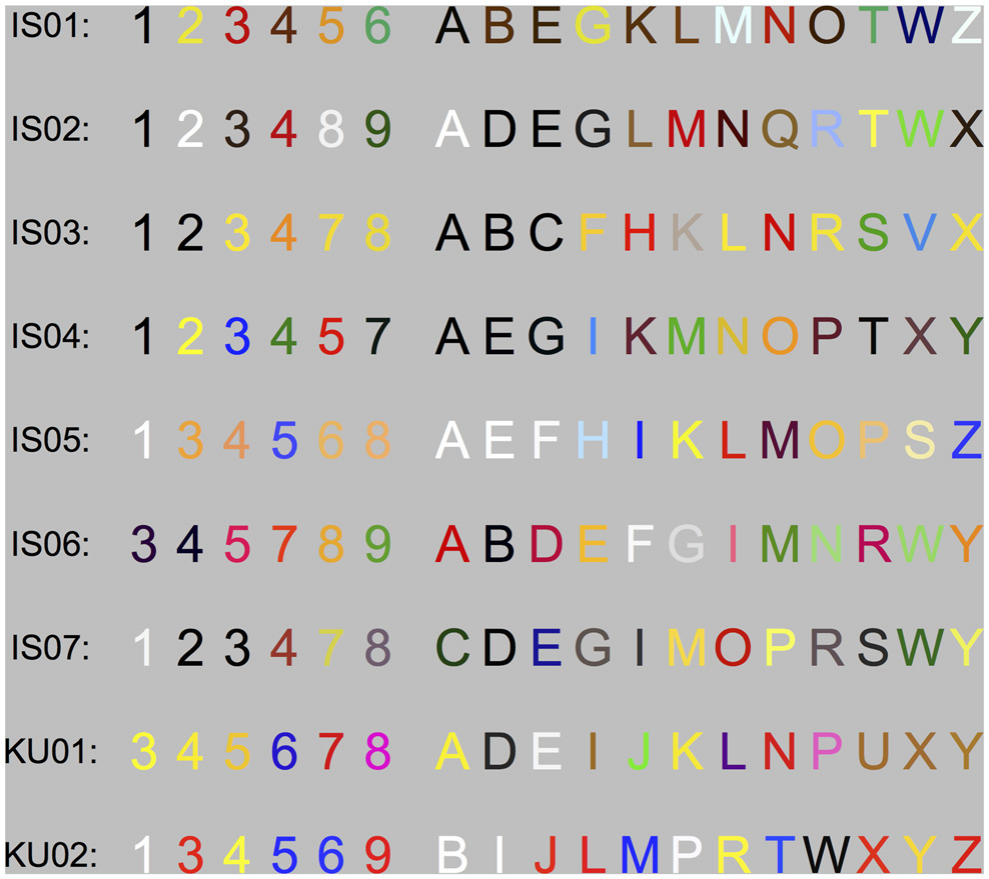
The experimental stimulus sets of each synesthete. Digits were used as distractors and letters as targets in experiment 1. Grapheme colors are averages (in RGB color space) over three trials of grapheme-color mapping on a computerized color palette (see Fig 3).

The experimental stimuli were Arial Bold graphemes; letter targets and digit distractors. Each grapheme was approximately 3.8° visual angle tall. The synesthetes were screened for grapheme-color synesthesia prior to the experiment and the experimental stimuli were customized based on data from this screening procedure (see *procedure* for details). For each synesthete-control observer pair, the graphemes were colored congruently with the synesthete’s grapheme-color association on half of the trials, while on the other half, they were colored with the inverse to the congruent color (incongruent condition). The identity of the letters varied by observer, but was always limited to 12 letters and 6 digits (see Fig 2). The letters were masked after presentation by rectangular pattern masks, consisting of 10 by 10 matrix of colored rectangles, combined randomly from the colors in given observer’s stimulus set. Inverse colors were defined in the RGB-color space as the separate R, G and B values, subtracted from the maximum bit value (255) of each color canon. Ten masks were generated for each pair of observers, and these were drawn randomly for each of the six positions on each trial.

Seven of the observers synesthetes were tested at the University of Iceland, Laboratory for Visual Perception and Visuomotor Control. These tests were carried out on a Dell desktop computer connected to a Compac s720 CRT display. The remaining two synesthetes and the nine controls were tested at the Center for Visual Cognition, University of Copenhagen. These tests were carried out on an Asus desktop computer connected to a ViewSonic CRT display. At both locations, displays had a 100 Hz refresh rate. Stimuli were presented with E-prime software and responses were made on standard USB-keyboards.

Physical measures of stimulus and background color and luminance were performed with a *Cambridge Research Systems* ColorCalII chromatic photometer, using the CRS toolbox for Matlab. Weber-contrast was the measure by using the equation (*I*_*s*_ − *I*_*b*_)/*I*_*b*_, where *I*_*s*_ and *I*_*b*_ represent the luminance intensity of the stimulus and the background, respectively.

### Procedure

#### Screening

**Fig 3:**
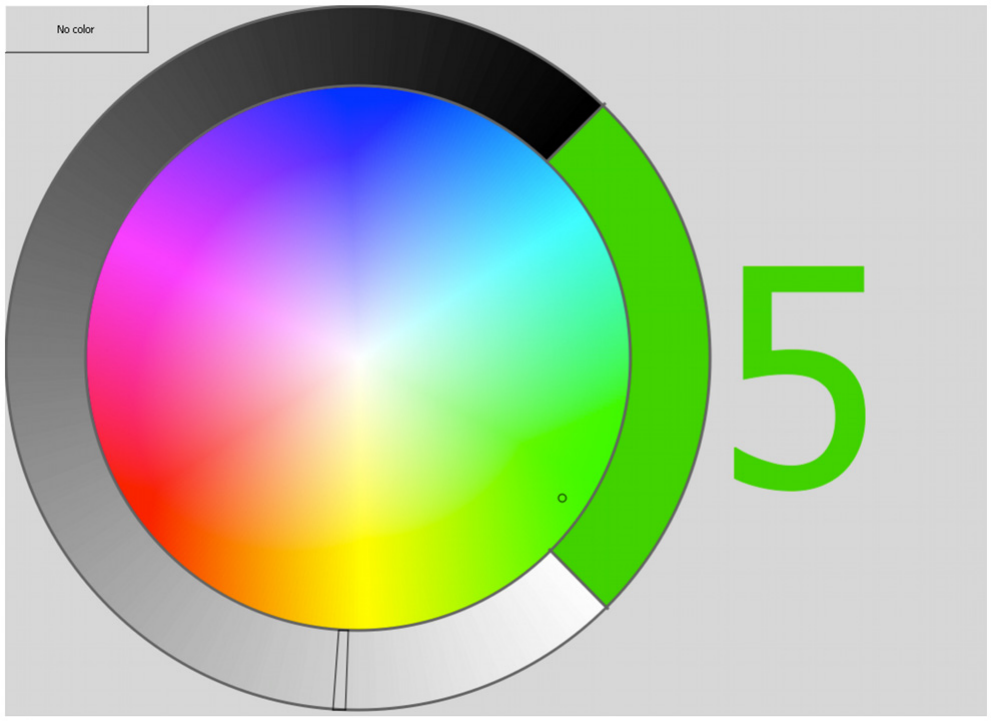
A screen shot from the grapheme-color mapping program used for the screening procedure of the study. Observers adjusted the hue of the right colored area on the annulus by navigating within the color palette. They adjusted the brightness by navigating the bright-to-dark area of the annulus. Navigation was controlled via the mouse pointer or keyboard, dependent on each observer’s preference.

Synesthete participants colored large typeface graphemes by means of a custom-made circular color palette, with adjustable luminance (Fig 3). Observers were presented with all the graphemes of the English alphabet and digits from 1–9 in random order. Each grapheme was presented 3 times, and observers made adjustments to the color and luminance to best match their synesthetic concurrents (see [71] for a similar procedure). If they did not have a synesthetic experience, they could select a “no color” button. After each registration of a grapheme-color the color palette would rotate randomly between 50° and 200°. This ensured that observers could not rely on spatial memory to make consistent grapheme-color mappings.

A consistency score was calculated for each grapheme, based on the three presentations of each grapheme. The consistency score was adopted from Eagleman and colleagues (see [71], Eq 1). Our criterion for inclusion in the test phase of the study was an average consistency score less than 1, as proposed by Eagleman. For practical purposes, we also required synesthetes to consistently map a minimum of 12 letters and 6 digits (a score less than 1 for each individual grapheme), after the exclusion of gray graphemes. Gray grapheme mappings were discarded due to the similarity with the background (*r* = 190, *g* = 190, *b* = 190). Four observers reported no color concurrent for some graphemes. Three of those reported no color for 2 graphemes, and the fourth for 7 graphemes.

**Fig 4:**
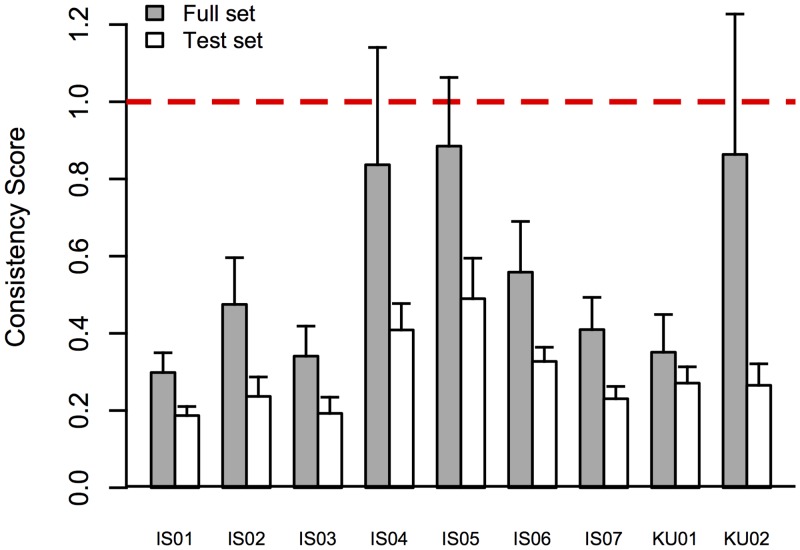
Consistency scores for all observers. The gray bars show the consistency for the full set of mapped graphemes (letters A–Z; digits 1–9). White bars show consistency scores for the 12 letters and 6 digits used in the experiment. The error bars represent +1 SEM. The red dashed line shows the inclusion criterion for participation in the experiment (see [71], Eq 1).

The results of the grapheme-color matching procedure were used to calculate the consistency of the synesthetes’ color experiences and the average color of each grapheme. Observers reported color concurrents for 28–35 (mean 33.6 graphemes). The average consistency score (see [71] Eq 1) for the 9 synesthetes, before the exclusion of gray graphemes, was .56 (*sd* = .24) and ranged from .30 for the most consistent observer to .89 for the least consistent one. All observers were sufficiently consistent on average to be included in the study. The grapheme set used in the experiment consisted of the 12 most consistent letters and 6 most consistent digits for each observer, after the exclusion of gray graphemes. For this subset, the average consistency score was .29 (*sd* = .10) and ranged from .18 to .48. Fig 4 shows the average consistency of each synesthete, for the full- and test-set of graphemes. The averaged colors for all test-set stimuli are shown in Fig 2.

#### Whole and Partial Report of Briefly Presented Graphemes

**Fig 5:**
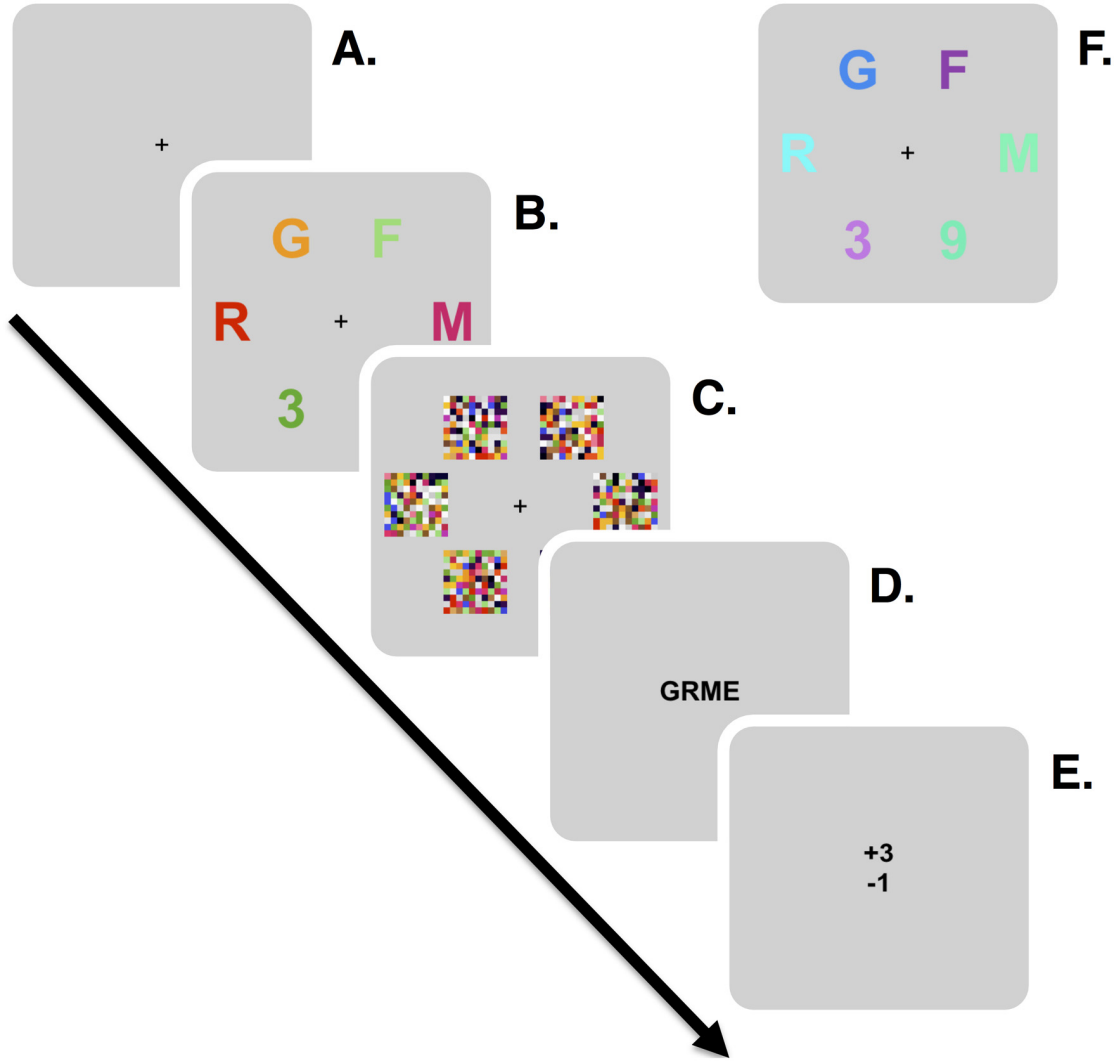
An illustration of the basic procedure in experiment 1. **A.** A fixation cross was presented until keystroke. **B.** Stimuli were presented for 10–150 ms. **C.** Stimuli were masked by pattern masks for 500 ms. **D.** Masks disappeared and observers responded by pressing the appropriate keys on a keyboard. Their responses were echoed in the center of the screen. **E.** When an observer had reported all letters from memory they pressed “Enter” and received instant feedback about their performance. **F.** An example of an incongruently colored grapheme display in a 4 target and 2 distractor condition.

The purpose of the study was to measure the attentional parameters proposed by TVA [33] and how these differ when processing synesthesia congruent, compared to incongruent, graphemes. We designed a mixed whole and partial report design [60, 62, 69], where up to six targets (letters) were presented with or without distractor elements (digits) for brief periods. Each observer would complete 4 blocks of 270 trials, 1080 trials in total. All elements in the display were either congruent or incongruent with the observers’ reported synesthetic concurrents. There were three combinations of targets and distractors: 6 targets with no distractors (6T0D), 4 targets with 2 distractors (4T2D) and 3 targets with 3 distractors (3T3D). These were presented for 10, 20, 30, 50, 80 or 150 ms, followed by a 500 ms pattern mask display. All the independent variables were chosen pseudo-randomly without replacement, so that all the conditions (exposure durations, congruence and target-distractor ratios) were balanced within each block of trials. Observers were instructed to report as many of the letters as possible, while ignoring the digits. If the observers did not recognize any letters, they could omit responses by by pressing the “Enter” key. They were also asked to keep uninformed guessing to a minimum, and strive towards an accuracy of 80–90% correct responses. Here, accuracy refers only to the successful reporting of typed-in responses, while omitted responses do not affect accuracy (for similar response criterion see [62, 72]). Every 36 trials, observers received feedback on their performance and were automatically prompted to be more conservative/liberal if their responses were below/above this level of accuracy. Fig 5 illustrates a single trial of the experiment, showing a typical 4T2D synesthesia congruent partial report trial (Fig 5B) and its incongruent counterpart (Fig 5F).

### Estimation of Attention Parameters

Two separate analyses were run for each subject, one estimating parameters for the synesthesia congruent trials and another for the incongruent trials. The individual data were fitted by to maximize the negative likelihood function, using the *LibTVA toolbox* for MatLab [73]. The model used to fit the data (on a trial by trial basis) assumes a total of 15 free parameters. The threshold of visual perception was drawn from a normal distribution with a mean and a standard deviation (2 free parameters; see [73], p. 419–420, for a precise mathematical description of the *t*_0_-parameter), speed of processing (*C*; 1 free parameter), the efficiency of visual selection was set as a ratio between the attentional weight of a target over a distractor (*α*, 1 free parameter), and VSTM (*K*) was drawn from a free probability distribution (5 free parameters; see [73], p. 418–419, for a precise mathematical description of the *K*-parameter). Finally, we estimated the relative attentional weights on the six possible spatial locations (5 free parameters) and compensated with a certain lapse probability (1 free parameter). Our analysis was focused on four TVA-parameters: 1) *t*_0_, the threshold of perception, defined as the longest ineffective exposure duration; 2) *C*, the total speed of processing, measured in graphemes/s; 3) *α*, the efficiency of visual selection, defined as the ratio between the attentional weight of a distractor compared to that of a target; 4) *K*, the capacity of VSTM measured in number of graphemes.

## Results

To test for a between-group difference in processing by congruent vs. incongruent stimulus sets, we analyzed the raw performance of the observers by a repeated measures ANOVA. We defined 3 factors in this analysis: *group* (controls vs. synesthetes) × *congruence of stimulus set* (congruent vs. incongruent) × *exposure duration* (from 10–150 ms) and tested whether they affected the probabilities of correctly reporting any given target in the display. The analysis did not reveal a main effect of group (*P*(*correct*)_*syn*._ − *P*(*correct*)_*control*_ = .016; *F*(1, 8) = .266, *p* = .62), suggesting that one group was not performing better than the other. There was a significant main effect of congruence (*P*(*correct*)_*congr*._ − *P*(*correct*)_*incongr*._ = .03; *F*(1, 8) = 12.012, *p* = .008), suggesting that the stimulus sets in the congruent condition were processed more efficiently than those from the incongruent condition, perhaps due to a slight difference in average contrast or more recognizable colors. Finally, and most importantly, there was a significant interaction between group and congruence, demonstrating that the synesthetes’ performance was more strongly affected by congruence with grapheme-color synesthesia (*F*(1, 8) = 5.782, *p* = .041). A congruent stimulus set boosted the synesthetes’ performance by 3.9 percentage points on average, compared to the incongruent conditions, while the control groups performance was enhanced by 2.1 percentage points. We repeated this analysis with average Weber-contrast difference (*absWC*_*conga*._ − *absWC*_*incongr*._) of each synesthete-control participant pair included as a covariate. This reduced the strength of the main effect of congruence (*F*(1, 8) = 8.223, *p* = .024), but enhanced the *group* × *congruence* interaction (*F*(1, 8) = 7.856, *p* = .026), supporting the hypothesis that the main effect of congruence was partly due differences in stimulus contrast.

The average contrast difference for each synesthete-control pair was measured with photometer. The difference between absolute Weber-contrast values in the congruent vs. incongruent stimulus sets was 10.6 percentage points on average (sd = 21.3 pp).

**Fig 6:**
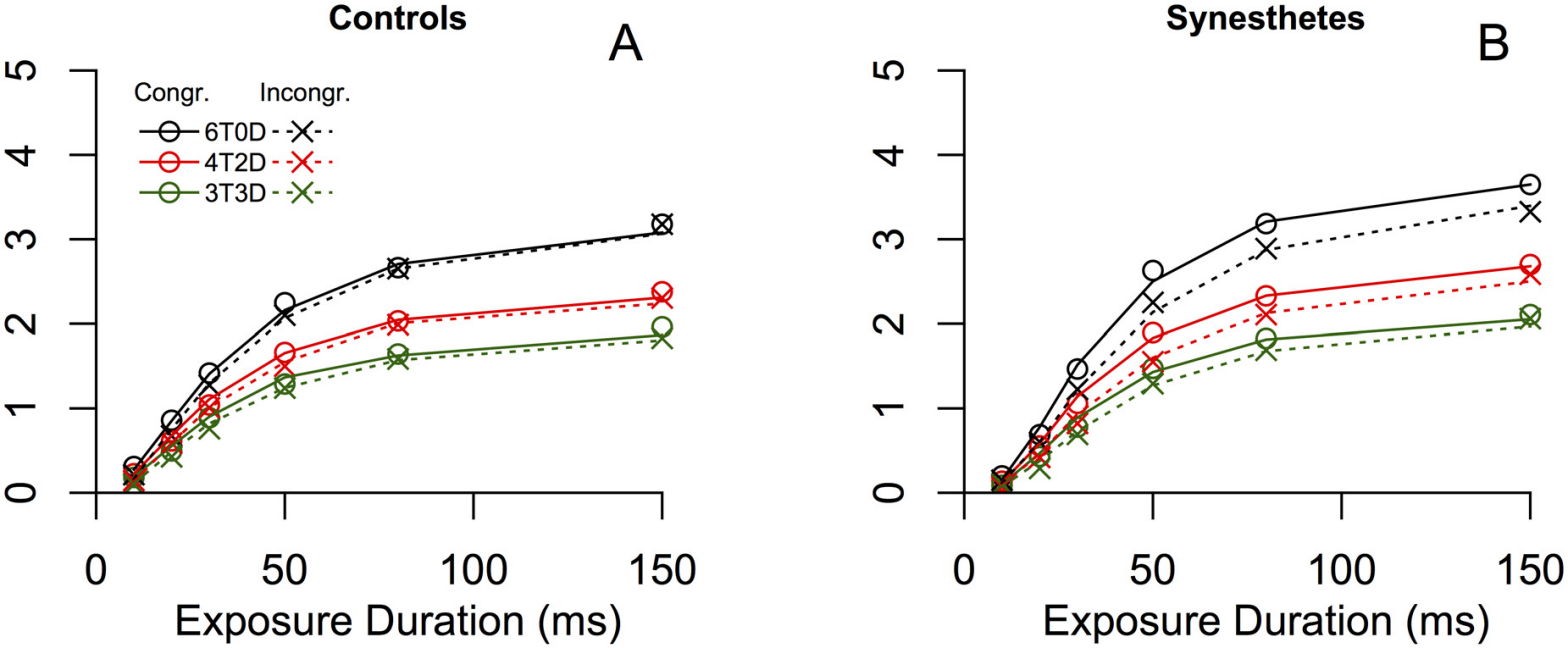
Average observed and modeled performance by target-to-distractor ratios as a function of exposure durations. Congruent trial performance is denoted by ∘ and incongruent performance by ×. TVA-fits for congruent trials are shown by the solid lines and fits to incongruent trials, by the dashed lines. Colors denote the ratio of targets and distractors in the briefly presented displays; black: 6 targets and no distractors, red: 4 targets and 2 distractors, green: 3 targets and 3 distractors. **A.** The results and fits by TVA of the control participants. **B.** The results and fits by TVA of the synesthetes.

Having established a conditional difference in colored grapheme processing of the two groups, we fitted TVA-models to the data, to obtain a richer understanding of the underlying attentional parameters. Fig 6 shows the average performance of controls and synesthetes, and the averaged psychometric functions fitted to the data by TVA. We ran repeated measures ANOVAs for each of the parameters independently, with the factors *group* × *congruence*. For the parameters that are theoretically dependent on stimulus contrast, we used the *absolute contrast difference* as a covariate.

### The threshold for visual perception (*t*_0_)

The threshold for visual perception is theoretically dependent on the strength of sensory evidence. If contrast is low, *t*_0_ will be higher than under high contrast conditions. We correlated the contrast difference between the congruent and incongruent stimulus sets with the differences in *t*_0_ between the two conditions. Significant correlations in the synesthete (*r* = .823, *p* = .006) and control group (*r* = .681, *p* = .043) corroborated the theoretical link between contrast and the threshold for perception.

A repeated measures ANOVA with *t*_0_ estimates as the dependent variable, the factors *group* × *congruence*, and *absolute contrast difference* as a covariate, did not reveal significant main effects of group (*p* = .516) or congruence (*p* = .42), nor did it demonstrate and interaction between the two factors (*p* = .468). Finally, a paired *t*-tests on the within-group congruence differences did not reveal any difference by congruence (see Table 1).

**Table 1:** Average parameter estimates by group (standard deviations in parentheses).

Group	parameter	congruent	incongruent	*t*	*df*	*p*
Controls	*t*_0_	9.35 (5.92)	9.46 (5.47)	-.133	8	.897
	*C*	91.69 (28.7)	82.67 (21.3)	1.346	8	.215
	*K*	3.23 (1.02)	3.22 (.922)	.064	8	.950
	*α*	.451 (.228)	.531 (.219)	-1.403	8	.198
Synesthetes	*t*_0_	11.8 (6.50)	12.7 (6.85)	-1.100	8	.303
	*C*	103.6 (35.1)	83.8 (25.3)	4.152	8	.003
	*K*	3.78 (.708)	3.55 (.597)	3.736	8	.006
	*α*	.570 (.217)	.538 (.183)	.466	8	.654

### The speed of grapheme processing (*C*)

Processing speed has a necessary relationship with contrast in TVA. The outcomes from the rate equation in TVA (see S1 Text) are dependent the sensory evidence (*η*(*x*, *i*)) for categorizations of visual stimuli. Therefore, TVA predicts higher stimulus contrast to result in faster visual processing. This theoretical prediction was corroborated by a significant correlation between contrast difference and *C*-parameter difference in the control group (*r* = .875, *p* = .002), while the correlation was lower and more not significant in the group of synesthetes (*r* = .378, *p* = .316).

A repeated measures ANOVA with *C* estimates as the dependent variable, the factors *group* × *congruence*, and *absolute contrast difference* as a covariate, did not reveal significant main effects of group (*p* = .506), but a marginally significant effect of congruence (*p* = .067). Crucially, the interaction between the two factors was significant (*F*(1, 8) = 11.968, *p* = .011), accounting for the larger conditional differences in processing speed in synesthetes, compared to the control group. Finally, paired *t*-tests of within-subject effects revealed a significant effect of congruence in the synesthete group (*t*(8) = 4.152, *p* = .003) but not in the control group (*t*(8) = 1.346, *p* = .215; see Table 1).

### Visual short-term memory capacity (*K*)

VSTM does not have direct theoretical links with stimulus contrast, assuming there is sufficient time to process the stimulus in question. Therefore, we did not include contrast difference as a covariate in the analysis of the *K*-parameter. A repeated measures ANOVA with the *K*-parameter as the dependent variable and the factors *group* × *congruence* did not reveal significant main effects of group (*p* = .206), congruence (*p* = .104) or the interaction between the two (*p* = .136). Yet, the trend in VSTM capacity estimates was consistently in the direction of a positive effect of congruence in the group of synesthetes (8 out of 9 participants), but not in the control group (4 out of 9 participants). Paired *t*-tests of within-subjects effects demonstrate a significant effect of congruence in the synesthete group (*t*(8) = 3.736, *p* = .006) but not in the control group (*t*(8) = .064, *p* = .95). The latter analysis suggests that we have insufficient statistical power to confirm a *group* × *congruence* interaction effect, but as Table 1 shows, there is almost no difference in *K*-capacity in the control group, while all but one synesthete has an enhanced VSTM capacity for congruent, according to the TVA-models.

### Attentional selectivity (*α*)

Selectivity is related to contrast in TVA, assuming that the evidence (*η*(*x*, *j*)) provided by the contrast helps the observer discriminate between targets and distractors (see the *weight equation* in S1 Text). A repeated measures ANOVA with *α* estimates as the dependent variable, the factors *group* × *congruence*, and *absolute contrast difference* as a covariate did not reveal any main effects of group (*F*(1, 8) = .001, *p* = .98), congruence (*F*(1, 8) = 2.555, *p* = .154), or an interaction between the two (*F*(1, 8) = .161, *p* = .7). Paired *t*-tests did not reveal any difference within-subjects (see Table 1).

Our failure to find main effects of group in any of our analyses strongly suggests that the interaction effects revealed in the aforementioned paragraphs were not due to differences in cognitive abilities. The parameters estimated to reveal specific components of attentional processing seem to be equivalent in the two groups, whether processing speed, visual memory, selectivity or perceptual threshold. Performance only diverges when congruence with synesthesia is taken into account.

## Discussion

In line with previous research, synesthetes performed better when processing congruently colored graphemes, while the lack of effects in the control group clearly demonstrates that the effects in our synesthetes are, in fact, related to congruence with their synesthetic experiences and not driven by the physical attributes of the presented stimuli. Congruence effects on performance have been demonstrated for colored grapheme processing before, but only in response time tasks where it is difficult to separate perceptual from post-perceptual processing and motor components. We found a perceptual processing advantage for congruent graphemes, and demonstrated that congruence with synesthetic concurrents can affect cognition at pre-categorical processing stages. However, the current results do not exclude post-perceptual effects of color congruence, which may well affect behavior when performing response time tasks, and task with more room for higher-order cognition. For example, it is quite possible that conceptual incongruence contributes to Stroop-interference. Here, we have established that synesthesia influences the perceptual stage(s) of colored grapheme processing, but not necessarily that it does not affect later stages as well.

The models fitted suggest that congruency dependent performance differences are due to a higher processing speed when processing congruent, compared to incongruent, graphemes. Within the theoretical framework of TVA, a likely cause is the long-term integration of synesthetic colors into perceptual categories; over time concurrent color information will be stored as predictive evidence and integrated into the grapheme category representation in VLTM. Therefore, the combination of a color and a grapheme would also be considered evidence for—or against—making a certain grapheme categorization. Thus, the congruent condition presents more evidence in the encoding race compared to the incongruent condition. Recent research on categorical visual search, where observers were prompted with a category and reported the presence or absence of a member of the prompted category in a search array, demonstrate that the typicality of a target can guide attention [74]. In the study, responses were faster and fixations more accurate, when a target was highly typical of the currently active search category. This line of evidence fits very well with both the theoretical framework of TVA, and the data reported here. The synesthetic observers are set to detect and report as many letters as possible, and when those letters are very typical of their category, in terms of shape and color, they manage to process the stimuli faster and hereby perform better. The increase in processing speed seems to be general, rather than due to increased selectivity, because we did not find systematic differences in the *α*-parameters when discriminating congruent, compared to incongruent, graphemes.

The models also suggested that VSTM capacity may have been positively affected by color congruence in the synesthete group. Recently, a number of studies have been conducted in an effort to answer the question of whether synesthetes have a memory advantage by comparing groups of synesthetes to control groups in various memory tasks (see [75] for a review). Generally, synesthetes seem to have an advantage in several memory-demanding tasks, albeit more moderate than suggested by the case-reports of synesthetes with exceptional memory [34, 35]. This advantage seems to be most pronounced in the domain of the inducing stimuli (digits and letters in grapheme-color synesthesia) but may also be extended to the domain of the concurrent experience (e.g. color) [6]. Here, we specifically measured the capacity of the passive visual short-term memory store. A congruency-dependent difference in VSTM is predicted by the theoretical frame-work of TVA, as more efficient categorical representations seem to facilitate and modulate short-term memory capacity [45] (see also [44] for similar findings). Similar to the effects on speed of processing, this effect may be caused by prolonged associations between a grapheme inducer and its concurrent color, which effectively makes the synesthete an expert in that particular categorization (e.g. categorizing “A” and the color “red” together), causing similar memory advantages as does expertise in other domains [45].

It is important to emphasize that this study does not directly address the processing of achromatic graphemes, which make up the bulk of studies on synesthesia and attention (see [2] for a review). Our results suggest that there is a perceptual processing advantage for congruently colored letters, compared to incongruent ones, because the congruence-dependent advantages affect the processing of a stimulus before it is categorized. This result must, therefore, be indicative of synesthetic-associations affecting pre-categorical processes. This is not to say that it supports pre-categorical theories of the *subjective experience* of synesthesia. Unlike studies of the effects of concurrent colors on the processing of achromatic graphemes, the current experimental task did not rely on the induced subjective synesthetic experiences. The results are equally compatible with pre-categorical theories, such as the cross-activation theory, which proposes that local cross-activations from grapheme processing areas to color processing areas are responsible for synesthetic experience [18, 76], and post-categorical hypotheses, such as the disinhibited-feedback hypothesis, that posits that synesthetic experience arises only after categorization of an inducing grapheme [17, 77]. To make sense of the results in terms of either hypothesis, one must only assume the interpretation proposed here: that consistent synesthetic experiences are able to modify the categorical representations of graphemes in VLTM. Direct testing of the influence of synesthesia on categorical representation requires longitudinal studies of congruent and incongruent grapheme processing. It is plausible that such a study could reveal a gradual strengthening of congruence effects over time, which would support this hypothesis. A recent longitudinal study of children by Simner and Baines [78] shows that the consistency of grapheme-color associations to develops and strengthens over a relatively long time period during development. Combining this type of investigation with modern imaging and electrophysiological techniques may help shed light on the specific neural mechanisms underlying the development of synesthetic associations.

Previous studies have demonstrated congruency-dependent effects on attention in synesthetes (e.g. using the Stroop-task). However, these types of studies have only revealed non-specific congruence effects on attentional processing. Here, we have measured *specific* components of attention in adults with synesthesia, and demonstrated—for the first time—how grapheme-color co-occurrences affect separate core elements of attentional processing in synesthetes; namely the speed of grapheme encoding (*C*) and, possibly also the capacity of VSTM (*K*). While other sub-components like the threshold for visual perception (*t*_0_) and selectivity (*alpha*) remained unaffected.

## Supporting Information

S1 Text A Detailed Explanation of TVA in the Context of the Experimental Task. (PDF)

S1 Dataset A compressed folder (.zip) containing the data from the whole and partial report of colored graphemes. The data structure is explained in the file *README.rtf*.
(ZIP)

## References

[1] Jewanski J, Day SA, Ward J. A Colorful Albino: The First Documented Case of Synaesthesia, by Georg Tobias Ludwig Sachs in 1812. J Hist Neurosci. 2009;18:293–303. 20183209 10.1080/09647040802431946

[2] Mattingley JB. Attention, Automaticity, and Awareness in Synesthesia. Ann N Y Acad Sci. 2009;1156(1):141–167. 10.1111/j.1749-6632.2009.04422.x 19338507

[3] Price MC. Synesthesia, Imagery and Performance In: Simner J, Hubbard EM, editors. Oxford Handbook of Synesthesia. New York: Oxford University Press; 2013 p. 728–757.

[4] Mulvenna CM. Synesthesia and creativity In: Simner J, Hubbard EM, editors. Oxford Handbook of Synesthesia. New York: Oxford University Press; 2013 p. 607–325.

[5] Meier B, Rothen N. Grapheme-color synaesthesia is associated with a distinct cognitive style. Front Psychol. 2013;4(September):1–7. 24065938 10.3389/fpsyg.2013.00632 PMC3777024

[6] Meier B, Rothen N. Synesthesia and Memory In: Simner J, Hubbard EM, editors. Oxford Handbook of Synesthesia. New York: Oxford University Press; 2013 p. 692–706.

[7] Edquist J, Rich AN, Brinkman C, Mattingley JB. Do synaesthetic colours act as unique features in visual search? Cortex. 2006;42(2):222–231. 16683496 10.1016/s0010-9452(08)70347-2

[8] Laeng B, Svartdal F, Oelmann H. Does color synesthesia pose a paradox for early-selection theories of attention? Psychol Sci. 2004;15(4):277–281. 10.1111/j.0956-7976.2004.00666.x 15043648

[9] Laeng B. Searching through synaesthetic colors. Atten Percept & Psychophys. 2009 9;71(7):1461–1467. 10.3758/APP.71.7.1461 19801606

[10] Mattingley JB, Payne JM, Rich AN. Attentional load attenuates synaesthetic priming effects in grapheme-colour synaesthesia. Cortex. 2006;42(2):213–221. 10.1016/S0010-9452(08)70346-0 16683495

[11] Palmeri TJ, Blake R, Marois R, Flanery MA, Whetsell W Jr. The perceptual reality of synesthetic colors. Proc Natl Acad Sci U S A. 2002;99(6):4127–4131. 10.1073/pnas.022049399 11904456 PMC122659

[12] Ramachandran VS, Hubbard EM. Psychophysical investigations into the neural basis of synaesthesia. Proc R Soc B Biol Sci. 2001;268(1470):979–983. 10.1098/rspb.2000.1576 PMC1088697 11370973

[13] Rich AN, Karstoft KI. Exploring the benefit of synaesthetic colours: Testing for “pop-out” in individuals with grapheme–colour synaesthesia. Cogn Neuropsychol. 2013;30(2):110–125. 10.1080/02643294.2013.805686 23768150 PMC3877912

[14] Smilek D, Dixon MJ, Cudahy C, Merikle PM. Synaesthetic photisms influence visual perception. J Cogn Neurosci. 2001;13(7):930–936. 10.1162/089892901753165845 11595096

[15] Ward J, Jonas C, Dienes Z, Seth A. Grapheme-colour synaesthesia improves detection of embedded shapes, but without pre-attentive ‘pop-out’ of synaesthetic colour. Proc Biol Sci. 2010;277(1684):1021–1026. 10.1098/rspb.2009.1765 20007177 PMC2842757

[16] Sagiv N, Heer J, Robertson L. Does binding of synesthetic color to the evoking grapheme require attention? Cortex. 2006;42:232–242. 16683497 10.1016/s0010-9452(08)70348-4

[17] Volberg G, Karmann A, Birkner S, Greenlee MW. Short- and long-range neural synchrony in grapheme-color synesthesia. J Cogn Neurosci. 2013;25:1148–62. 10.1162/jocn_a_00374 23448520

[18] Brang D, Hubbard EM, Coulson S, Huang M, Ramachandran VS. Magnetoencephalography reveals early activation of V4 in grapheme-color synesthesia. Neuroimage. 2010;53:268–274. 10.1016/j.neuroimage.2010.06.008 20547226

[19] Rouw R, Scholte HS. Increased structural connectivity in grapheme-color synesthesia. Nat Neurosci. 2007;10(6):792–797. 10.1038/nn1906 17515901

[20] Stroop JR. Studies of interference in serial verbal reactions. J Exp Psychol. 1935;18(6):643–662. 10.1037/h0054651

[21] Mattingley JB, Rich AN, Yelland G, Bradshaw JL. Unconscious priming eliminates automatic binding of colour and alphanumeric form in synaesthesia. Nature. 2001;410(6828):580–582. 10.1038/35069062 11279495

[22] Colizoli O, Murre JM, Rouw R. Pseudo-synesthesia through reading books with colored letters. PloS one. 2012;7(6):e39799 10.1371/journal.pone.0039799 22761905 PMC3384588

[23] Dixon MJ, Smilek D, Cudahy C, Merikle PM. Five plus two equals yellow. Nature. 2000;406(6794):365 10.1038/35019148 10935623

[24] Mills CB, Boteler EH, Oliver GK. Digit synaesthesia: A case study using a Stroop-type test. Cognitive Neuropsych. 1999;16(2):181–191. 10.1080/026432999380951

[25] Lupiáñez J, Callejas A. Automatic Perception and Synaesthesia: Evidence from Colour and Photism Naming in a Stroop-Negative Priming Task. Cortex. 2006;42(2):204–212. 10.1016/S0010-9452(08)70345-9 16683494

[26] Monahan JS. Coloring single Stroop elements: Reducing automaticity or slowing color processing? J Gen Psychol. 2001;128(1):98–112. 10.1080/00221300109598901 11277451

[27] Stirling N. Stroop interference: An input and an output phenomenon. Q J Exp Psychol. 1979;31(1):121–132. 10.1080/14640747908400712

[28] Lamers MJ, Roelofs A, Rabeling-Keus IM. Selective attention and response set in the Stroop task. Mem cognition. 2010;38(7):893–904. 10.3758/MC.38.7.893 20921102

[29] Vendrell P, Junqué C, Pujol J, Jurado M, Molet J, Grafman J. The role of prefrontal regions in the Stroop task. Neuropsychologia. 1995;33(3):341–352. 10.1016/0028-3932(94)00116-7 7792000

[30] MacLeod CM. Half a century of research on the Stroop effect: an integrative review. Psychol bull. 1991;109(2):163 10.1037/0033-2909.109.2.163 2034749

[31] Rich AN, Mattingley JB. Can attention modulate color-graphemic synesthesia? In: Robertson L, Sagiv N, editors. Synesthesia: Perspectives from Cognitive Neuroscience: Perspectives from Cognitive Neuroscience. Oxford University Press; 2004 p. 108.

[32] Luck SJ, Vogel EK. The capacity of visual working memory for features and conjunctions. Nature. 1997;390(6657):279–281. 10.1038/36846 9384378

[33] Bundesen C. A theory of visual attention. Psychol Rev. 1990;97(4):523–547. 10.1037/0033-295X.97.4.523 2247540

[34] Luria AR. The Mind of a Mnemonist: A Little Book about a Vast Memory Penguin modern psychology. Harvard University Press; 1968.

[35] Baron-Cohen S, Bor D, Billington J, Asher J, Wheelwright S, Ashwin C. Savant memory in a man with colour form-number synaesthesia and asperger. J Consciousness Stud. 2007;14(9–10):237–251.

[36] Ericsson KA, Lehmann AC. Expert and exceptional performance: Evidence of maximal adaptation to task constraints. Annu Rev Psychol. 1996;47(1):273–305. 10.1146/annurev.psych.47.1.273 15012483

[37] Yaro C, Ward J. Searching for Shereshevskii: What is superior about the memory of synaesthetes? Q J Exp Psychol. 2007;60(5):681–695. 10.1080/17470210600785208 17455076

[38] Rothen N, Meier B. Grapheme-colour synaesthesia yields an ordinary rather than extraordinary memory advantage: evidence from a group study. Memory. 2010;18(3):258–264. 10.1080/09658210903527308 20169501

[39] Pritchard J, Rothen N, Coolbear D, Ward J. Enhanced associative memory for colour (but not shape or location) in synaesthesia. Cognition. 2013;127(2):230–234. 10.1016/j.cognition.2012.12.012 23454796

[40] Witthoft N, Winawer J. Synesthetic Colors Determined by Having Colored Refrigerator Magnets in Childhood. Cortex. 2006;42(2):175–183. 10.1016/S0010-9452(08)70342-3 16683491

[41] Witthoft N, Winawer J. Learning, Memory, and Synesthesia. Psychol Sci. 2013;24(3):258–265. 10.1177/0956797612452573 23307940 PMC3648671

[42] Brang D, Ramachandran VS. Survival of the Synesthesia Gene: Why Do People Hear Colors and Taste Words? PLoS Biol. 2011 11;9(11):e1001205 10.1371/journal.pbio.1001205 22131906 PMC3222625

[43] Witthoft N, Winawer J, Eagleman DM. Prevalence of Learned Grapheme-Color Pairings in a Large Online Sample of Synesthetes. PLoS One. 2015;10:e0118996 10.1371/journal.pone.0118996 25739095 PMC4349591

[44] Curby KM, Glazek K, Gauthier I. A visual short-term memory advantage for objects of expertise. J Exp Psychol Hum Percept Perform. 2009;35(1):94 10.1037/0096-1523.35.1.94 19170473 PMC4159943

[45] Sørensen TA, Kyllingsbæk S. Short-Term storage capacity for visual objects depends on expertise. Acta Psychol (Amst). 2012;140(2):158–163. 10.1016/j.actpsy.2012.04.004 22627160

[46] Cowan N, Ricker TJ, Clark KM, Hinrichs GA, Glass BA. Knowledge cannot explain the developmental growth of working memory capacity. Dev Sci. in press;. 10.1111/desc.12197 PMC4270959 24942111

[47] Bundesen C, Habekost T. Principles of Visual Attention: Linking Mind and Brain Oxford Psychology Series. New York: Oxford University Press; 2008.

[48] Bundesen C. Visual attention: Race models for selection from multielement displays. Psych Res. 1987;49(2–3):113–121. 10.1007/BF00308676 3671629

[49] Zhang W, Luck SJ. Discrete fixed-resolution representations in visual working memory. Nature. 2008;453(7192):233–235. 10.1038/nature06860 18385672 PMC2588137

[50] Zhang W, Luck S. Capacity & Resolution Trade Off in Iconic Memory but not in Working Memory. J Vis. 2011 9;11(11):1248 10.1167/11.11.1248

[51] Cowan N. The magical number 4 in short-term memory: a reconsideration of mental storage capacity. Behav Brain Sci. 2001;24:87–114. 10.1017/S0140525X01003922 11515286

[52] Bundesen C, Habekost T, Kyllingsbaek S. A Neural Theory of Visual Attention: Bridging Cognition and Neurophysiology. Psychol Rev. 2005;112(2):291–328. 10.1037/0033-295X.112.2.291 15783288

[53] Chun MM. Visual working memory as visual attention sustained internally over time. Neuropsychologia. 2011;49(6):1407–1409. 10.1016/j.neuropsychologia.2011.01.029 21295047

[54] Alvarez GA, Cavanagh P. The Capacity of Visual Short-Term Memory is Set Both by Visual Information Load and by Number of Objects. Psychol Sci. 2004;15(2):106–111. 10.1111/j.0963-7214.2004.01502006.x 14738517

[55] Bays PM, Catalao RFG, Husain M. The precision of visual working memory is set by allocation of a shared resource. J Vis. 2009;9(10):1–11. 10.1167/9.10.7 19810788 PMC3118422

[56] Baddeley A. Working memory. Science (80-). 1992;255(5044):556 10.1126/science.1736359 1736359

[57] Aben B, Stapert S, Blokland A. About the distinction between working memory and short-term memory. Front Psychol. 2012;3(August):1–9. 22936922 10.3389/fpsyg.2012.00301 PMC3425965

[58] Rothen N, Meier B. Do synesthetes have a general advantage in visual search and episodic memory? A case for group studies. PLoS One. 2009;4(4). 10.1371/journal.pone.0005037 PMC2660420 19352425

[59] Terhune DB, Wudarczyk OA, Kochuparampil P, Kadosh RC. Enhanced dimension-specific visual working memory in grapheme–color synesthesia. Cognition. 2013;129(1):123–137. 10.1016/j.cognition.2013.06.009 23892185 PMC3757159

[60] Shibuya H, Bundesen C. Visual selection from multielement displays: measuring and modeling effects of exposure duration. J Exp Psychol Hum Percept Perform. 1988;14(4):591–600. 10.1037/0096-1523.14.4.591 2974870

[61] Smith PL, Lee YE, Wolfgang BJ, Ratcliff R. Attention and the detection of masked radial frequency patterns: Data and model. Vision Res. 2009;49(10):1363–1377. 10.1016/j.visres.2008.04.024 18538812

[62] Sø rensen TA, Vangkilde S, Bundesen C. Components of Attention Modulated by Temporal Expectation. J Exp Psychol Learn Mem Cogn. 2014;Available from: http://www.ncbi.nlm.nih.gov/pubmed/25068851. 10.1037/a0037268 25068851

[63] Vangkilde S, Bundesen C, Coull JT. Prompt but inefficient: Nicotine differentially modulates discrete components of attention. Psychopharmacology (Berl). 2011;p. 1–14. 10.1007/s00213-011-2361-x PMC3222829 21629997

[64] Townsend J. Theoretical analysis of an alphabetic confusion matrix. Percept Psychophys. 1971;9(1):40–50. 10.3758/BF03213026

[65] Pelli DG, Burns CW, Farell B, Moore-Page DC. Feature detection and letter identification. Vis res. 2006;46(28):4646–4674. 10.1016/j.visres.2006.04.023 16808957

[66] Wolfe JM, Horowitz TS. What attributes guide the deployment of visual attention and how do they do it? Nat Rev Neurosci. 2004;5(6):495–501. 15152199 10.1038/nrn1411

[67] Nordfang M, Dyrholm M, Bundesen C. Identifying bottom-up and top-down components of attentional weight by experimental analysis and computational modeling. J Exp Psychol Gen. 2013;142(2):510–535. 10.1037/a0029631 22889161

[68] Petersen A, Andersen TS. The effect of exposure duration on visual character identification in single, whole, and partial report. J Exp Psychol Hum Percept Perform. 2012;38(2):498–514. 10.1037/a0026728 22288689

[69] Sperling G. The Information Available in Brief Visual Presentations. Psychol Monogr. 1960;74:1–29. 10.1037/h0093759

[70] Ásgeirsson AG, Kristjánsson A, Bundesen C. Independent priming of location and color in identification of briefly presented letters. Atten Percept Psychophys. 2014;76(1):40–8. 10.3758/s13414-013-0546-6 24092356

[71] Eagleman DM, Kagan AD, Nelson SS, Sagaram D, Sarma AK. A standardized test battery for the study of synesthesia. J Neurosci Methods. 2007;159(1):139–145. 10.1016/j.jneumeth.2006.07.012 16919755 PMC4118597

[72] Habekost T, Rostrup E. Visual attention capacity after right hemisphere lesions. Neuropsychologia. 2007;45(7):1474–1488. 10.1016/j.neuropsychologia.2006.11.006 17174988

[73] Dyrholm M, Kyllingsbaek S, Espeseth T, Bundesen C. Generalizing parametric models by introducing trial-by-trial parameter variability: The case of TVA. J Math Psychol. 2011;55:416–429. 10.1016/j.jmp.2011.08.005

[74] Maxfield JT, Stalder WD, Zelinsky GJ. Effects of target typicality on categorical search. J Vis. 2014;14(12):1–11. 10.1167/14.12.1 25274990 PMC4181372

[75] Rothen N, Meier B, Ward J. Enhanced memory ability: Insights from synaesthesia. Neurosci Biobehav Rev. 2012 9;36(8):1952–1963. 10.1016/j.neubiorev.2012.05.004 22634573

[76] Hubbard EM, Brang D, Ramachandran VS. The cross-activation theory at 10. J Neuropsychol. 2011 9;5(2):152–177. 10.1111/j.1748-6653.2011.02014.x 21923784

[77] Grossenbacher PG, Lovelace CT. Mechanisms of synesthesia: Cognitive and physiological constraints. Trends Cogn Sci. 2001;5(1):36–41. 10.1016/S1364-6613(00)01571-0 11164734

[78] Simner J, Bain AE. A longitudinal study of grapheme-color synesthesia in childhood: 6/7 years to 10/11 years. Front Hum Neurosci. 2013;7 10.3389/fnhum.2013.00603 PMC3826064 24312035

