# Supplementary material for: Components of Attention in Grapheme-Color Synesthesia: A Modeling Approach
Source: PLoS One. 2015 Aug 7;10(8):e0134456. doi: 10.1371/journal.pone.0134456 (PMC4529240; doi:10.1371/journal.pone.0134456)
Supplement: S1 Text — (PDF) [file pone.0134456.s001.pdf]

A DETAILED EXPLANATION OF TVA IN THE CONTEXT OF THE EXPERIMENTAL TASK

The central equations of TVA

According to TVA, the rates of processing for each visual element are the result of two central equations. The first is the *weight equation*, which yields the relative attentional weight of each element in the visual field:

$$w_x = \sum_{j \in R} \eta(x, j)\pi_j \tag{1}$$

where w_x is the attentional weight of element x, π_j is the pertinence of feature j, $\eta(x, j)$ is the evidence for element x having feature j and R is the set of all perceptual features. This is the *filtering* mechanism of TVA, and it represents the top-down control of selective attention.

The second mechanism needed to categorize perceptual elements in TVA is *pigeonholing*; the designation of an element into a certain category. In the current context, the most important perceptual categories are the response categories; i.e. the identities of letters. The evidence for an object x belonging to such a category is multiplied by a *bias* (β_i) which is a quantification of an observers‘ bias towards making certain categorizations. The filtering and pigeonholing mechanisms of TVA are implemented object-wise throughout the visual field yields a *rate of encoding* toward all categorizations for each object in a scene, based on prior knowledge (η; evidence values from memory templates), pertinence (π), and bias (β). The rates (v) of an element x being recognized in VSTM as belonging to category x is given by the second central equation: the *rate equation*:

$$v(x, i) = \eta(x, i)\beta_i \frac{w_x}{\sum\limits_{z \in S} w_z} \tag{2}$$

A DETAILED EXPLANATION OF TVA IN THE CONTEXT OF THE EXPERIMENTAL TASK

The route of visual information processing according to TVA.

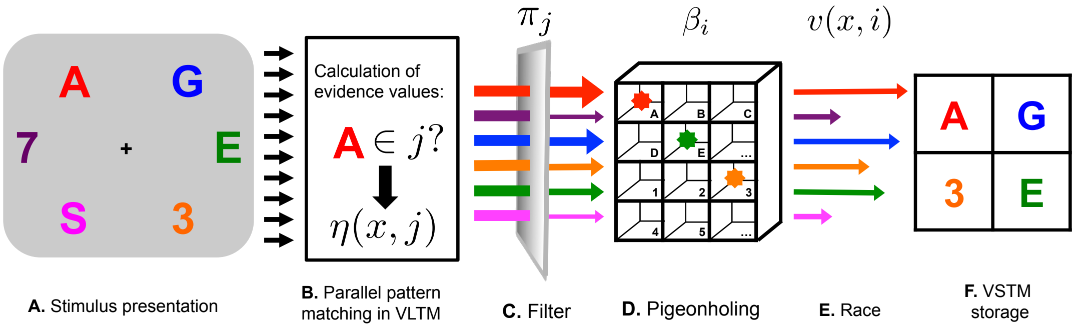

Here, we illustrate the process of TVA as it theoretically unfolds during a trial of the current experiment. **A.** The observer is presented with a circular array of 6 graphemes; 2 of which are distractors (digits) and 4 are targets (letters). **B.** The first process is consists of a massive parallel comparisons of sensory input to perceptual category representations in VLTM. This process yields the evidence values for each elements probability of belonging to any given perceptual category. **C.** The evidence for an element belonging to the target category (letters in the current example) will determine how efficiently it is filtered. Here, the sensory evidence, weighted by its pertinence (see equation 1), is represented by the thickness of the arrows subsequent to filtering **D.** The filtered sensory evidence is pigeonholed into perceptual categories; here shown as the parts of the response categories for the experimental task. The probability of a visual element x being categorized as belonging to category i is weighted by the bias (β) for making that particular categorization. **E.** The product of the filtering and pigeonholing mechanisms determines the rate ($v(x,i)$) of each element x in a stochastic exponential race towards recognition in a capacity limited VSTM store (see equation 2). Each element races towards a categorization with the rate represented by the length of the arrows. The higher the rate, the more likely it is that the observer makes that particular categorization **E.** When a categorization "wins" a race, it is represented in VSTM and, consequently, available to conscious report. The capacity of VSTM is usually limited to 3–5 visual elements. In this example, the observer has a capacity of 4 visual elements and has managed to fill all 4 "slots" with the targets A, G and E, and, inadvertently, the distractor 3.
