# Supplementary material for: Components of Attention in Grapheme-Color Synesthesia: A Modeling Approach
Source: PLoS One. 2015 Aug 7;10(8):e0134456. doi: 10.1371/journal.pone.0134456 (PMC4529240; doi:10.1371/journal.pone.0134456)
Supplement: S1 Dataset — The data structure is explained in the file README.rtf. (ZIP) [file pone.0134456.s002.zip › README.rtf]

The data-files included in this folder as support material to a paper by Asgeirsson, Nordfang and Sorensen, is formatted for fitting with the libTVA toolbox (Dyrholm et al., 2011). The first line of each file gives the number of trials. The first column (after line 1) is an arbitrary condition number. The second column is the exposure duration of each trial in milliseconds. The third column shows the targets for a each trial. Zeros represent positions with no targets. The order of letters/zeros shows the position of each target, counting from the NE (upper right) position on a 6 position imaginary circle, and going clockwise to the NW-position (upper left). The fourth columns shows the distractors and their positions, ordered in the same way as the targets. The fifth column shows the responses for each trial. Filenames that start with two capital letters; e.g. IS01, represent the experimental group of the study, where the two letter prefix is the test location (IS for Iceland, KU for Copenhagen), while the number represents the order in which each subject entered the study. Filenames that have a lower case “c” before a location prefix represent the control group. The Location prefix and number for the control participants, represents what stimulus set they were exposed to. Questions to:arnigunnarasgeirsson@gmail.comÁrni G. Ásgeirsson, November, 2014. 
